# Science as momentary truth^☆^

**DOI:** 10.1016/j.bjorl.2017.09.006

**Published:** 2017-10-19

**Authors:** Leonardo Silva

**Affiliations:** Faculdade de Ciências Médicas da Santa Casa de São Paulo (FCMSCSP), Departamento de Otorrinolaringologia, São Paulo, SP, Brazil

Dear Editor,

A recent BJORL editorial touched on a sensitive issue related to Continuing Medical Education (CME).^1^ How can we physicians reconcile our increasingly scarce time with the rapid advances in medicine? Voluminous, easily accessible and sometimes contradictory scientific information has made CME a controversial subject throughout the world. What to study, how to study and in what form to do it? What is the best database? Where are the best studies? Where to find the right answer to a specific question? How to separate high-quality scientific articles from low-quality publications?

It is worth remembering that when starting from a position of doubt, only the scientific method has the power to guide the researcher safely in the search for the best answer. Understanding this point is fundamental for separating science from belief, and nothing is more important for any study.

Undoubtedly one of the greatest advances in the organization and diffusion of medical knowledge has been the advent of Evidence-Based Medicine (EBM).^2^ EBM centers around the world, including McMaster in Canada and Oxford in England, are dedicated to develop tools that provide the physician with synthetic and simple scientific outputs with hierarchical qualification, that allow quick access to relevant information and with low cost. Among these tools are systematic reviews with meta-analysis.

When accessing secondary sources, we focus directly on what really matters because the separation of wheat from the shaft has already been done. This does not mean that studies with a higher level of evidence should escape the attentive and critical screening of the reader.

Criticisms of MBE exist and are many. Science and truth, however, cannot be considered static and immutable; they obviously run into the epistemological limits of the stage at which the evolution of human knowledge finds itself. Thus, we can point out as an example that even meta-analyses produced in the sixteenth century attesting to geocentrism were correct. It is clear that the tool should not be confused with the finished product. The “scientific truth” of that moment was unraveled by the substrate the data held at the time, like a photo. To deny this would be to deny the very evolution of human knowledge.

By understanding, valuing and disseminating MBE, we contribute to the efficiency of health services that ultimately depend directly on the production, transmission and application of quality knowledge.

## Conflicts of interest

The author declares no conflicts of interest.
